# Variability in the Duration and Thoroughness of Hand Hygiene

**DOI:** 10.1093/cid/ciz612

**Published:** 2019-09-13

**Authors:** Joel M Mumma, Francis T Durso, Lisa M Casanova, Kimberly Erukunuakpor, Colleen S Kraft, Susan M Ray, Andi L Shane, Victoria L Walsh, Puja Y Shah, Craig Zimring, Jennifer DuBose, Jesse T Jacob

**Affiliations:** 1 School of Psychology, Georgia Institute of Technology, Atlanta; 2 School of Public Health, Georgia State University, Atlanta; 3 Division of Infectious Diseases, Department of Medicine, Emory University School of Medicine, Atlanta; 4 Department of Pathology and Laboratory Medicine, Emory University School of Medicine, Atlanta; 5 Division of Infectious Diseases, Department of Pediatrics, Emory University School of Medicine and Children’s Healthcare of Atlanta, Atlanta; 6 School of Architecture, Georgia Institute of Technology, Atlanta

**Keywords:** hand hygiene, human factors engineering, personal protective equipment, serious communicable disease

## Abstract

We observed 354 hand hygiene instances across 41 healthcare workers doffing personal protective equipment at 4 hospital-based biocontainment units. We measured the duration and thoroughness of each hand hygiene instance. Both parameters varied substantially, with systematic differences between hospitals and differences between healthcare workers accounting for much of the variance.

## Teaser

Using observations of hand hygiene practices during simulated patient care in biocontainment units, we partitioned variance in the duration and thoroughness of hand hygiene into that attributable to differences between facilities and differences between healthcare workers.

Hand hygiene is a cornerstone of infection prevention. In the setting of serious communicable diseases such as Ebola virus disease, healthcare workers (HCWs) perform hand hygiene frequently while doffing personal protective equipment (PPE) to limit the transfer of contamination. Effective hand hygiene requires appropriate contact time (duration) and coverage of surfaces (thoroughness) [1], which should be achieved consistently each time hand hygiene is performed. However, our previous observations [2] of HCWs doffing high-level PPE in different biocontainment units suggest that these parameters may vary substantially in practice because of a lack of standardization in how hand hygiene is performed across healthcare facilities (allowing differences in the protocols of facilities to contribute to variability) as well as a lack of mechanisms for ensuring that HCWs adhere to specific hand hygiene techniques (allowing differences between individual HCWs to contribute to variability). Using our previous observations of hand hygiene practices during simulated patient care in biocontainment units, in the present study, we partitioned variance in the duration and thoroughness of hand hygiene into that which was uniquely attributable to facilities and to HCWs.

## METHODS

We performed a retrospective analysis of the hand hygiene practices of 41 HCWs during simulations at 4 state-designated Ebola treatment centers (sites A–D) in Georgia. In each simulation, a single HCW donned high-level PPE, performed a standardized clinical task, and then doffed his or her PPE according to institutional protocol [2]. Ten HCWs participated in the simulations at each facility (11 at site A). Most of the participating HCWs were nurses (90%), with the remainder including paramedics (5%) and HCWs with other roles (5%). All simulations involved a trained observer (TO) who used a written checklist to guide each HCW through his or her facility’s doffing protocol. At 2 facilities, the same individual served as the TO for either all or nearly all (90%) of the simulations.

Simulations were video-recorded using 1 handheld camera and between 2 and 5 stationary cameras, which were later used to determine the duration and thoroughness of each hand hygiene instance. The duration of a hand hygiene instance was defined as the total elapsed time between the moment a HCW began rubbing his or her hands with a hand hygiene product until the moment his or her hands came apart to begin the next doffing step. The thoroughness of a hand hygiene instance was defined as the percentage of 6 surfaces across both hands (ie, wrists, thumbs, and in between all fingers) that were visibly rubbed during hand hygiene.

Hierarchical linear models were created using the lme4 package in R statistical software [3]. A 3-level random intercept model was constructed separately for the duration of hand hygiene and for the thoroughness of hand hygiene. In both models, the lowest-level units were individual hand hygiene instances (n = 354), which were nested within HCWs (middle level units; n = 41), who were nested within facilities (highest level units; n = 4). The statistical significance of the variance of each random intercept (ie, the variance between facilities and the variance between HCWs) was assessed with the lmerTest package [4]. A *P* value of <.05 was considered statistically significant. Intraclass correlation coefficients (ICCs), which can be interpreted as the proportion of total variance in an outcome that is attributable to the grouping structure in the population [5], were calculated for the random intercept of facilities and of HCWs.

## RESULTS

Two facilities (A and D) used alcohol-based hand rub (ABHR) exclusively for hand hygiene, whereas site B used ABHR for all but the first instance of hand hygiene, for which they used a disinfecting wipe. Site C used disinfecting wipes for hand hygiene predominantly, using ABHR only once after the inner pair of gloves was removed. Site D used automatic alcohol dispensers exclusively for hand hygiene and enforced the duration of hand hygiene by having both the TO and healthcare worker sing “Happy Birthday” aloud. The median number of hand hygiene instances per HCW was similar among facilities, ranging from 7 (interquartile range [IQR], 6–7) to 11.5 (IQR, 10–13).

**Figure 1.**
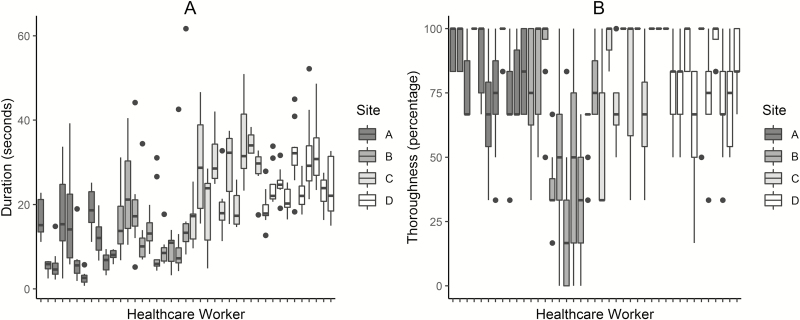
Box plots of the duration (*A*) and thoroughness (*B*) of hand hygiene instances of 41 healthcare workers (HCWs) across 4 Ebola treatment centers. Each box plot corresponds to the hand hygiene instances of a single HCW in a facility.

The median duration of hand hygiene at each facility ranged from 7.3 (IQR, 4.2–14.9) to 25.5 (IQR, 17.5–32.2) seconds. Among the 41 HCWs (Figure 1A), the median duration of hand hygiene was 17.4 seconds (IQR, 12.1–23.9). The ICCs from the hierarchical linear model (see Supplementary Table 1) suggest that 61% of the total variance in the duration of hand hygiene was attributable to systematic differences between facilities and HCWs, with differences between facilities (42%, *P* < .001) accounting for more than double that attributable to differences between HCWs (19%, *P* < .001). Last, we observed TOs occasionally truncating hand hygiene by moving the HCW on to the next doffing step, although this was only observed at 2 facilities, affecting 11% and 23% of all hand hygiene instances.

Across all 354 hand hygiene instances, the most commonly missed surfaces were thumbs (38%), followed by wrists (25%) and in between fingers (17%). The median thoroughness of hand hygiene among the 4 facilities ranged from 67% (IQR, 33%–83%) to 100% (IQR, 67%–100%). Among the 41 HCWs (Figure 1B), the median thoroughness of hand hygiene was 83% (IQR, 67%–100%). The ICCs from the hierarchical linear model (see Supplementary Table 2) suggest that 51% of the total variance in the thoroughness of hand hygiene was attributable to differences between facilities and HCWs, with differences between facilities (18%, *P* = .009) accounting for approximately half that attributable to differences between HCWs (33%, *P* < .001).

## DISCUSSION

In our sample of 41 trained HCWs at 4 state-designated Ebola treatment centers, we observed substantial variability in both the duration and thoroughness of hand hygiene while doffing high-level PPE for simulated patients with serious communicable diseases. More than half of the variance in duration and thoroughness was attributable to systematic differences between facilities and between HCWs. Regarding duration, differences between facilities accounted for approximately twice as much variance as did differences between HCWs. Indeed, we observed large differences between the facilities’ protocols for hand hygiene; for example, 1 facility consistently enforced the duration of hand hygiene by using a song (site D), whereas another facility used disinfectant wipes predominantly (site C) rather than ABHR. Regarding thoroughness, differences between facilities accounted for approximately half as much variance as did differences between HCWs. The smaller contribution of facilities aligns with our observation that no facility gave explicit direction on the thoroughness of hand hygiene in their protocol. Otherwise, variance in the duration or thoroughness of hand hygiene due to differences between HCWs may be explained by characteristics of HCWs, which we did not measure in the present study; for example, HCW workload and preexisting attitudes toward hand hygiene have been identified as risk factors for nonadherence [6].

In the high-stakes environment of biocontainment units, variability in the duration and thoroughness of hand hygiene should be reduced by standardizing how hand hygiene is performed across facilities as well as by standardizing the mechanisms for ensuring that HCWs adhere to specific hand hygiene techniques throughout doffing. Regarding the former, consensus guidelines exist for both adequate duration and coverage of surfaces, although the exact values of these parameters have been debated [1, 7]. Regarding the latter, mechanisms that minimize individual differences between HCWs during hand hygiene are essential and might include standardizing how HCWs are trained to perform hand hygiene (including knowledge, skills, and attitudes toward hand hygiene) [1], increasing the TO’s role in enforcing the duration and thoroughness of hand hygiene (eg, by incorporating written instructions for both duration and thoroughness into a facility’s protocol), and using features of the built environment for regulating adequate duration and thoroughness (eg, a timer or visual cues such as posters) [1, 8].

A similar need for the standardization of hand hygiene practices exists in common clinical settings, where hand hygiene is typically monitored through direct observation using “secret shoppers,” product consumption, or electronic systems without the benefit of a TO monitoring performance [1]. In general, these methods focus on compliance, consistent with the World Health Organization’s (WHO’s) “Five Moments of Hand Hygiene” campaign [9], and capture when HCWs perform hand hygiene, but not necessarily the quality of their hand hygiene. Because duration and thoroughness are not routinely measured by the WHO’s “Five Moments of Hand Hygiene,” standardization of hand hygiene practices beyond compliance with opportunities for hand hygiene remains even more challenging than overall adherence. Integrating measures of duration and thoroughness to current measures of hand hygiene would be onerous in many clinical settings. However, successful implementation of electronic hand hygiene systems may allow redirection of existing hand hygiene resources for visual observation away from compliance to quality [10]. Whether in a biocontainment unit or on a ward, priority should be placed not only on when hand hygiene is performed but also on the quality of hand hygiene.

## Supplementary Data

Supplementary materials are available at *Clinical Infectious Diseases* online. Consisting of data provided by the authors to benefit the reader, the posted materials are not copyedited and are the sole responsibility of the authors, so questions or comments should be addressed to the corresponding author.

ciz612_suppl_Supplementary_Information

## Notes

***Acknowledgments*.** The authors thank the participating healthcare workers, hospitals, and team members of the Prevention Epicenter of Emory and Atlanta Consortium Hospitals.

***Financial support*.** This work was supported by the Prevention Epicenters Program of the Centers for Disease Control and Prevention (award U54CK000164). Emory University’s participation was supported in part by the National Center for Advancing Translational Sciences of the National Institutes of Health (award UL1TR000454; Atlanta Clinical and Translational Science Institute).

***Supplement sponsorship.*** This article appears as part of the supplement “Personal Protective Equipment for Preventing Contact Transmission of Pathogens: Innovations from CDC’s Prevention Epicenters Program,” sponsored by the CDC’s Prevention Epicenters Program.

***Potential conflicts of interest.*** All authors report no potential conflicts. All authors have submitted the ICMJE Form for Disclosure of Potential Conflicts of Interest. Conflicts that the editors consider relevant to the content of the manuscript have been disclosed.
