# Supplementary material for: Variability in the Duration and Thoroughness of Hand Hygiene
Source: Clin Infect Dis. 2019 Sep 13;69(Suppl 3):S221–3. doi: 10.1093/cid/ciz612 (PMC6743505; doi:10.1093/cid/ciz612)
Supplement: ciz612_suppl_Supplementary_Information [file ciz612_suppl_supplementary_information.docx]

**Supplementary Table 1.** Estimates of fixed and random effects (i.e., random intercepts) for the duration of hand hygiene.

| **Fixed Effect** | **Coefficient** | **SE** | ***P*** | **ICC** |
| --- | --- | --- | --- | --- |
| Intercept | 19.07 | 3.9 | .016 | -- |
| **Random Effects** | **Variance Component** | **SD** |  |  |
| Facility | 57.65 | 7.59 | < .001 | .42 |
| Healthcare Worker | 26.70 | 5.17 | < .001 | .19 |

*Note*. SE = Standard error. SD = Standard deviation. ICC = Intraclass correlation coefficient.

**Supplementary Table 2.** Estimates of fixed and random effects (i.e., random intercepts) for the thoroughness of hand hygiene.

| **Fixed Effect** | **Coefficient** | **SE** | ***P*** | **ICC** |
| --- | --- | --- | --- | --- |
| Intercept | 0.77 | 0.06 | .001 | -- |
| **Random Effects** | **Variance Component** | **SD** |  |  |
| Facility | 0.01 | 0.11 | .009 | .18 |
| Healthcare Worker | 0.02 | 0.15 | < .001 | .33 |

*Note*. SE = Standard error. SD = Standard deviation. ICC = Intraclass correlation coefficient.
